# Breast cancer in relation to induced abortions in a cohort of Chinese women

**DOI:** 10.1038/sj.bjc.6600603

**Published:** 2002-10-21

**Authors:** Z Ye, D L Gao, Q Qin, R M Ray, D B Thomas

**Affiliations:** Fred Hutchinson Cancer Research Center, 1100 Fairview Ave N, PO Box 19024,MP 474, Seattle, Washington, WA 98109-1024, USA; The Station for Prevention and Treatment of Cancer of the Shanghai Textile Industry Bureau, 1474 Yan An Xi Road, Shanghai 200052, Peoples Republic of China

**Keywords:** breast cancer, induced abortion, epidemiology

## Abstract

The possible influence of induced abortion on breast cancer risk was assessed in a cohort of 267 040 women enrolled in a randomised trial of breast self-examination in Shanghai, China. Based on answers to a baseline questionnaire, subsequent breast cancer risk was not significantly associated with ever having an induced abortion. After adjustment for potential confounders, the relative risk estimate was 1.06 (95% C.I.: 0.91, 1.25), and there was no trend in risk with number of abortions. Analysis of data from more detailed interviews of 652 cases and 694 controls from the cohort yielded similar results. There was also no overall increase in risk in women with induced abortion after first birth. Few women had undergone an abortion after 13 weeks gestation or before their first child. Although increases in risk were observed in such women, they were not statistically significant and could have been due to recall bias. Abortions as they have been performed in China are not an important cause of breast cancer.

*British Journal of Cancer* (2002) **87**, 977–981. doi:10.1038/sj.bjc.6600603 www.bjcancer.com

© 2002 Cancer Research UK

## 

During the early months of a pregnancy, the mammary epithelium undergoes massive proliferation and then this is followed by differentiation in preparation for lactation (Neville and Daniel, 1987; Norman and Litwack, 1987). It has been hypothesised that interruption of a pregnancy before differentiation occurs would increase the risk of breast cancer (Russo and Russo, 1980). Results of case–control studies of breast cancer in relation to induced abortions are inconsistent, as are conclusions from reviews of this topic (Brind *et al*, 1996; Wingo *et al*, 1997; Bartholomew and Grimes, 1998; Davidson, 2001). No cohort studies (Melbye *et al*, 1997; Lindefors-Harris *et al*, 1989; Lazovich *et al*, 2000), or case–control studies nested within cohorts with ascertainment of abortion prior to development of breast cancer (Newcomb and Mandelson, 2000; Tang *et al*, 2000), have shown associations of breast cancer with induced abortions, suggesting that some results of the case–control studies may have been due to differential reporting of induced abortions by cases and controls.

Induced abortions have been widely used in China since the 1970s as part of the national family planning programme, aimed at achieving the goal of one child per family. Abortions are performed primarily on married women to limit family size. These are socially acceptable and readily reported (Sanderson *et al*, 2001). Those few abortions performed prior to the delivery of a living child tend to be before marriage, are therefore less socially acceptable, and probably not as likely to be reported in an interview as abortions after marriage.

This investigation was conducted in a cohort of over a quarter of a million female textile workers in Shanghai who were interviewed and enrolled in a randomised trial of breast self-examination in 1989–91 (Thomas *et al*, 1997, 2002). It consists of two parts: a cohort study and a case–control study nested within the cohort, conducted to obtain additional information not available on the entire study population. This provided an unusual opportunity to estimate with high statistical power the relative risk of breast cancer in relation to several features of induced abortions, and to assess the possible influence of ascertainment bias on results based on the case–control approach.

## MATERIALS AND METHODS

### Subjects

#### The cohort study

The women in the cohort were those enrolled in a randomised trial of Breast Self-Examination (BSE) in female textile workers in Shanghai (Thomas *et al*, 1997, 2002). All current and retired women employed by the Shanghai Textile Industry Bureau (STIB), who were born between 1925 and 1958, and who were permanent residents of Shanghai, were eligible for the BSE Trial. Thirty-four nurses or former STIB factory medical workers were recruited and trained to be BSE field workers. These BSE workers, working with approximately 5000 factory medical workers, conducted all field operations. A census was carried out at the beginning of the study and a roster for recruitment of all potentially eligible women was developed. Baseline activities for the BSE study were conducted during the period from October 1989 to October 1991. A baseline questionnaire was administered to all women by the medical workers to obtain information on risk factors for breast cancer and other variables, including number of induced and spontaneous abortions, and the outcome of the first pregnancy.

There were 292 503 women listed on the original roster, and after eliminating women who were ineligible, had left the STIB by the time of the baseline activities, did not complete the baseline questionnaire, or who had breast cancer before the beginning of the BSE study, 267 040 women remained in the BSE trial, and constituted the population for this cohort study.

As part of the BSE trial, all new breast cancer cases were identified by factory medical workers at each factory. This was supplemented by checking the records of the STIB tumour and death registry, which passively receives reports of all incident cancer cases from each factory annually, and by periodic review of the records of the Shanghai Cancer Registry. When a case was identified, her name, age, diagnosis date, hospital, address and the BSE study identification number were obtained. From September 1, 1989 to August 31, 1995, 702 cases of histologically confirmed breast cancer were identified and included as incident cases for this study.

#### The case–control study

The same 702 incident cases of breast cancer were also eligible for this nested case–control study. Controls were randomly selected from the BSE cohort. They were frequency matched to the cases using 34 exact year of birth (1925–1958) categories. Twice the number of controls as the number of cases born in each calendar birth year was selected. The women with randomly assigned odd numbers were selected as primary controls and the women with even numbers were asked to participate if the attempt to access the first control failed. In all but 42 instances first controls were interviewed and included in the study.

Data collection was carried out between September 1, 1995 and February 28, 1996. Fourteen interviewers were recruited from retired female nurses at three hospitals affiliated with the STIB. Cases and controls were interviewed in person in the women's homes. The interview took approximately 1 h and requested detailed information on the major known and suspected risk factors for breast cancer. Detailed information on each abortion was collected, including the woman's age at time of the procedure, whether it was before or after other pregnancies, and weeks of gestation.

Informed consent was obtained from each woman prior to interview. The study was approved by the Institutional Review Boards of the Fred Hutchinson Cancer Research Center and the Station for Prevention and Treatment of Cancer of the Shanghai Textile Industry Bureau, in accordance with an assurance filed with the Office for the Protection from Research Risks (OPRR) of the National Institutes of Health.

### Data analysis

#### The cohort study

In exploratory analyses unconditional multiple logistic regression models were used to calculate odds ratios (and their 95% confidence intervals) as estimates of relative risk. To evaluate possible confounding factors, age plus individual risk factors were entered sequentially into logistic regression models as categorical variables, and those that altered the age adjusted relative risk estimate in relation to induced abortion by over 5% were included in the final model. The significance of trends in risk with level of exposure was calculated by assigning scores to the categorised levels of exposure and treating the scores as a continuous variable in the regression models. To evaluate possible interactions between induced abortion and other risk factors, the risk of breast cancer in relation to induced abortion was computed by stratified analysis. Likelihood ratio tests were performed to test interactions between induced abortion and all other variables included in the final analyses. The risk of breast cancer in relation to spontaneous abortion was also estimated after adjustment for potential confounding variables, using the same procedures as for induced abortion. Logistic regression models were used in order to facilitate direct comparisons of the odds ratios derived from the case–control study.

#### The case–control study

Odds ratios and 95% confidence intervals obtained from unconditional logistic regression models were used to estimate relative risks. Age was included in all logistic models. The same procedures that were used in the cohort study were employed to identify and control for potential confounding factors, and to test for trends and interactions. The risk of breast cancer in relation to spontaneous abortion was similarly estimated after adjustment for potential sources of confounding.

Some items in the case–control study questionnaire were comparable to those in the baseline questionnaire, except that they were obtained nearly 6 years later. To evaluate possible recall bias in the case–control study, the information collected for that study on history of induced abortion and other variables of interest was compared to the corresponding data obtained on the baseline questionnaire after accounting for possible differences due to the different times of data collection.

## RESULTS

### The cohort study

Compared to women having a first birth before age 20, nulliparous women had a significantly increased risk of breast cancer (RR=2.32, 95% CI: 1.45, 3.70) and there was a significant trend in risk with age at first birth. Risk was inversely related to number of live births and reduced in women who had ever breast fed (RR=0.79, 95% CI: 0.66, 0.96). Women who were post-menopausal at baseline were at slightly lower risk than pre-menopausal women of the same age. Risk was increased in women having a history of a breast lump (RR=2.39, 95% C.I.: 1.82, 3.15) and a family history of breast cancer (RR=1.65, 95% C.I.: 1.08, 2.54). No associations of risk were observed with: marital status, age at menarche, history of a stillbirth, tubal ligation, hysterectomy or oophorectomy, or use of oral or injectable contraceptives, an IUD, cigarettes, or alcohol.

After adjustment for age and age at first live birth, no other variable altered risk in relation to an induced abortion by more than 5% and only these variables were included in the final model. After adjustment for age, no other variable altered the relative risk in relation to a spontaneous abortion by more than 5%, but age at first birth was included in the final model to make it comparable to the model for the risk in relation to induced abortion.

As shown in Table 1

**Table 1:**
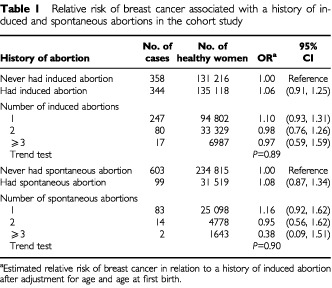
Relative risk of breast cancer associated with a history of induced and spontaneous abortions in the cohort study

, there was no increased risk in women who had ever undergone an induced abortion and no trend in risk with number of induced abortions. There were no nulliparous women with breast cancer who had an induced abortion, and the results were virtually unchanged when analyses were restricted to women who had ever been pregnant or ever had a full term pregnancy. Only four of 4318 women who reported that their first pregnancy was aborted developed breast cancer for a risk of 0.51 (95% CI: 0.31, 1.50) relative to women whose first pregnancy ended in a live birth. Risk was also not associated with a history of a spontaneous abortion, and there was no trend in risk with number of spontaneous abortions.

The relative risk estimates in women under the age of 50 years, and aged 50 years or over, were 1.02 (95% CI: 0.80, 1.29) and 1.07 (95% CI: 0.87, 1.31) for women with an induced abortion, and 1.31 (95%CI: 0.90, 1.92) and 1.00 (95% CI: 077, 1.29) for women with a spontaneous abortion, respectively. No significant interactions were observed between history of an induced abortion and any of the other variables considered.

### The case–control study

Of the 702 eligible cases identified in the cohort, 652 (93%) were interviewed, 36 cases were deceased, eight were not located, and six refused to be interviewed. Of the 744 selected controls, 694 (93%) were successfully interviewed, four were deceased, 38 were not located and eight refused to be interviewed.

Most of the associations with risk that were observed in the cohort study were also observed in the case–control study. These include relationships to nulliparity, age of birth of first child, number of live births, having ever breast fed, menopausal status, history of benign breast disease, and family history of breast cancer. In addition, risk was associated with several variables not available from the baseline questionnaire, including more than 12 years of schooling, body mass index at age 20 years and, unexpectedly, a first pregnancy ending in a stillbirth (RR=1.82, 95% CI: 1.06, 3.10). As with results based on the baseline questionnaire, risk as estimated from the case–control study was not significantly altered in relation to a history of ever having a stillbirth, tubal ligation, hysterectomy or oophorectomy, or use of oral or injectable contraceptive or an IUD, alcohol drinking, or history of smoking. It was also not associated with level of physical activity at work, or the smoking history of the woman's husband.

As shown in Table 2

**Table 2:**
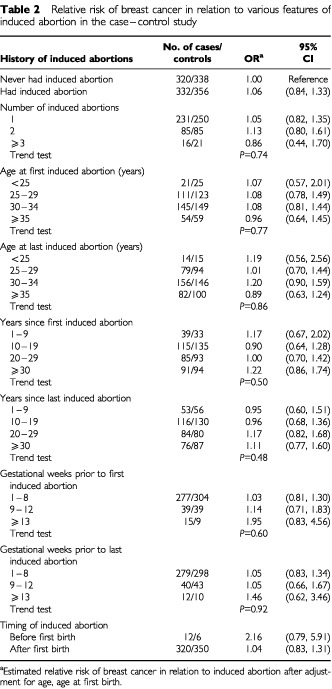
Relative risk of breast cancer in relation to various features of induced abortion in the case–control study

, there was no significant alteration in risk in relation to history of an induced abortion. Results similar to this finding were observed for women who had ever been pregnant or ever had a full term pregnancy. There were too few nulliparous women with an abortion (one case and no controls) to provide an estimate of the relative risk in such women.

There are no significant trends in risk with number of induced abortions, age at first or last induced abortion, or years since first or last abortion. Unlike results from the cohort study, there is a possible increased risk in relation to an induced abortion before a woman's first birth, but the relative risk estimate is based on small numbers of women and has a wide confidence interval. There is a non-significant increase in risk in relation to duration of gestation before either first or last induced abortion.

When the analysis was restricted to women aged under 50 years, no significant increases in risk in relation to induced abortion were evident, but a possible increased risk was seen in women having had an induced abortion before their first birth (RR=2.34, 95% CI: 0.78, 7.05) and in woman whose first (RR=1.92, 95% CI: 0.62, 5.98) or last (RR=1.33, 95% CI: 0.39, 4.54) induced abortion occurred after 13 weeks gestation. There were insufficient numbers of women age 50 years or over with induced abortions before their first birth for meaningful analysis. These women did have a non-significant increase in risk in relation to 13 or more weeks at gestation before first (RR=2.13, 95% CI: 0.58, 7.76) and last (RR=1.75, 95% CI: 0.52, 5.93) induced abortion.

Only seven cases and one control had their first abortion after 9 weeks and before their first birth, giving a risk of 7.38 (95% CI: 0.90, 60.83) relative to women with no induced abortion (320 cases and 338 controls). The relative risk for first abortion after 13 weeks and before first birth could not be estimated because of small numbers of exposed women. There was no significant alteration in risk in women having an induced abortion after their first birth regardless of length of gestation.

The relative risk of breast cancer in relation to an induced abortion in women was estimated separately in women with and without various risk factors for breast cancer, and no significant interactions between induced abortion and other variables were observed.

Women who had experienced a spontaneous abortion were not at increased risk (RR=0.81, 95% CI: 0.59, 1.12), either before or after the age of 50 years. Risk was also not increased in relation to a spontaneously aborted first pregnancy (RR=1.27; 95% CI: 0.51, 3.16).

### Comparisons between the cohort study and the case–control study

A comparison of data on induced abortion from the case–control and baseline questionnaires is shown in Table 3

**Table 3:**
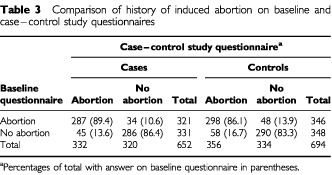
Comparison of history of induced abortion on baseline and case–control study questionnaires

. The overall agreement on the two questionnaires was 87.9% for cases and 84.7% for controls. The overall agreement on history of spontaneous abortion was 91.7 and 90.3% for cases and controls, respectively (not shown).

To assess the possible effect of recall bias on the results, the relative risk was estimated using various combinations of the data from the cohort and the case–control studies to define women who had ever undergone an induced abortion. These analyses included 652 cases and 694 controls shown in Table 3 that completed the relevant items on both questionnaires. The relative risk estimates were similar when induced abortion was defined as that reported from the case–control study questionnaire (RR=1.06; 95% CI: 0.84, 1.33), as reported on the baseline questionnaire (RR=1.03; 95% CI: 0.82, 1.29), as reported consistently on both questionnaires with women with inconsistent responses excluded from the analysis (RR=0.99; 95% CI: 0.78, 1.24), as reported on either one of the two questionnaires with women with inconsistent answers being considered to have had an abortion (RR=0.99; 95% CI: 0.78, 1.24), and as women with inconsistent responses being considered not to have had an abortion (RR=1.07; 95% CI: 0.84, 1.38). The same procedures were performed for estimating the risk in relation to spontaneous abortion, and the corresponding relative risk estimates were 0.81 (0.59, 1.12), 1.05 (0.77, 1.43), 0.99 (0.64, 1.36), 0.92 (0.70, 1.22), and 0.92 (0.63, 1.34) respectively.

## DISCUSSION

The present cohort study did not show an excess risk of breast cancer in relation to ever having an induced abortion, or in relation to number of such abortions, in women either under or over the age of 50 years.

Since the present study was conducted in a population with low risk of breast cancer, if an effect of an abortion on risk is additive the present study would have been more likely to have detected the effect than studies in high-risk populations.

The increase in risk in relation to induced abortion observed in most other studies was small (Davidson, 2001), and residual negative confounding could therefore explain the absence of such an association in this study. However, associations of risk with most established risk factors for breast cancer were observed and the relative risk of breast cancer in relation to induced abortion was not appreciably altered by adjustment for these factors, suggesting that residual confounding is not a likely explanation for our findings.

Incomplete information on induced abortion irrespective of the development of breast cancer is another possible explanation for the absence of a relationship between breast cancer and induced abortion in either the present cohort or case–control study. However, unlike previous studies, such as those carried out in the United States, where induced abortion was legalized only after 1970 and tends not to be socially acceptable, our investigations were conducted where induced abortion has been legal for over 50 years, encouraged by public policy and widely acceptable. Under such circumstances, under-reporting of most induced abortions would not likely be an important problem. In a population-based case–control study in Shanghai (in which no association between breast cancer and induced abortions was observed), 66% of the study participants resported having had an induced abortion (Sanderson *et al*, 2001); and about 60% of the women included in three surveys elsewhere in China reported having had an abortion. These percentages are somewhat higher than in the present study cohort (51%). Although this could reflect under-reporting of abortions by about 10–15%, the effect on the relative risk estimates would be small. Furthermore, this lower percentage with an abortion history may reflect superior access to contraceptive services by women employed in the textile industry, and hence fewer unplanned pregnancies leading to an abortion.

One meta-analysis of data from multiple studies resulted in a summary relative risk of 1.3 (95% CI: 1.2, 1.4) in women who had ever undergone an induced abortion (Brind *et al*, 1996). The present cohort study had 90% power to detect a true relative risk in women with a history of induced abortion as low as 1.1.

Differential reporting of induced abortions by cases and controls is a problem that has been identified in case–control studies of induced abortion and breast cancer in other countries (Lindefors-Harris *et al*, 1991). However, the agreement between the answers on the baseline and the case–control study questionnaires on history of an induced abortion in this study was moderately high, and similar for cases and controls, strongly suggesting that there was no bias in reporting most abortions in the present case–control study. This study thus supports the results of a prior population-based case–control study in urban Shanghai (Sanderson *et al*, 2001) that also found no significant alteration in risk of breast cancer in relation to induced abortions.

If induced abortion was related to poorly differentiated breast cancer, with a poor prognosis, deceased cases would be more likely to have a history of induced abortion than those who were interviewed, and the risk in relation to induced abortion would have been underestimated (Daling *et al*, 1996). To assess this possibility, the relative risk was estimated including all women eligible for the case–control study, but determining induced abortions from the baseline questionnaire, and no increased risk in relation to induced abortion was observed.

A full-term pregnancy first causes mammary cell proliferation and then differentiation, thus presumably reducing susceptibility to carcinogenesis. It is speculated that an early interruption of a pregnancy may lead to enhanced proliferation of breast tissue without subsequent differentiation, and hence to increased susceptibility to carcinogenic change. If this is true, then risk of breast cancer would be expected to increase with the gestational length prior to an abortion, and this effect might be particularly evident in women without a prior full term pregnancy. Compatible with this hypothesis and with a prior study in Denmark (Melbye *et al*, 1997) a non-significant increase in risk was observed in our case–control study in women who had undergone an induced abortion at gestational week 13 or later; and this apparent relationship was especially strong for abortions before a first birth. However, these findings were based on small numbers, were not statistically significant, and are not consistent with results from our cohort study which showed no increase in risk in women whose first pregnancy was aborted. They could be due to recall bias because an aborted first pregnancy in China would be more likely than abortions after a full term pregnancy to have been performed before marriage and hence not to have been as socially acceptable and faithfully reported as other abortions.

Although our study could not exclude the possibility of an increased risk in women having an induced abortion before their first birth and after a relatively long period of gestation, abortions in China are generally performed early and primarily to limit family size, and are not an important cause of breast cancer. They are not a likely explanation for the recent increase in rates of breast cancer in that country (Jin *et al*, 1993).
